# Assessing association between protein truncating variants and quantitative traits

**DOI:** 10.1093/bioinformatics/btt409

**Published:** 2013-07-16

**Authors:** Manuel A. Rivas, Matti Pirinen, Matthew J. Neville, Kyle J. Gaulton, Loukas Moutsianas, Cecilia M. Lindgren, Fredrik Karpe, Mark I. McCarthy, Peter Donnelly

**Affiliations:** ^1^Wellcome Trust Centre for Human Genetics, Nuffield Department of Medicine, University of Oxford, Oxford OX3 7BN, UK, ^2^Institute for Molecular Medicine Finland, University of Helsinki, Helsinki 00290, Finland, ^3^Oxford Centre for Diabetes, Endocrinology and Metabolism, Radcliffe Department of Medicine, Oxford OX3 7LJ, UK, ^4^NIHR Oxford Biomedical Research Centre, OUH Trust, Oxford OX3 7LE, UK and ^5^Department of Statistics, University of Oxford, Oxford OX1 3TG, UK

## Abstract

**Motivation:** In sequencing studies of common diseases and quantitative traits, power to test rare and low frequency variants individually is weak. To improve power, a common approach is to combine statistical evidence from several genetic variants in a region. Major challenges are how to do the combining and which statistical framework to use.

General approaches for testing association between rare variants and quantitative traits include aggregating genotypes and trait values, referred to as ‘collapsing’, or using a score-based variance component test. However, little attention has been paid to alternative models tailored for protein truncating variants. Recent studies have highlighted the important role that protein truncating variants, commonly referred to as ‘loss of function’ variants, may have on disease susceptibility and quantitative levels of biomarkers. We propose a Bayesian modelling framework for the analysis of protein truncating variants and quantitative traits.

**Results:** Our simulation results show that our models have an advantage over the commonly used methods. We apply our models to sequence and exome-array data and discover strong evidence of association between low plasma triglyceride levels and protein truncating variants at *APOC3* (Apolipoprotein C3).

**Availability:** Software is available from http://www.well.ox.ac.uk/~rivas/mamba

**Contact:** donnelly@well.ox.ac.uk

## 1 INTRODUCTION

Advances in DNA sequencing and customized exome arrays are quickly transforming the landscape of genomic studies of diseases and related quantitative traits (Bonnefond *et al.*, 2012; Jonsson *et al.*, 2012; Momozawa *et al.*, 2011; Nejentsev *et al.*, 2009; Rivas *et al.*, 2011). In sequencing studies of common diseases and quantitative traits, power to individually test any rare or low frequency variant is typically weak in attainable sample sizes. Hence, to boost the ability to detect signal, evidence is combined across variants. When

designing such an ‘aggregation’ test, there are three main questions to consider:

- Across which biological units should variants be combined?
- Which variants mapping within those units should be included?
- Which statistical models should be used?

As for the first question, an obvious choice of biological unit across which to combine variants is the gene, and we focus on this throughout the article, considering all the coding sequence of the gene as the relevant unit. An alternative would be to combine variants across all genes within a pathway, but this adds several complications, not least because of the currently rather imprecise knowledge of biological pathways.

Within a gene one could include all observed variants, but this would be likely to include many neutral variants and/or variants with opposite direction of effects, both of which can lead to loss of power under many statistical methods. The ideal approach would be to combine only those variants that affect the trait of interest. This is, however, difficult in practice because these will not be known in advance. One approximation would be to include only the variants with functional effects. Even this is challenging with current limited knowledge of the function of coding variants in the human genome: commonly used predictors of the function of non-synonymous variants (Adzhubei *et al.*, 2010; Kumar *et al.*, 2009) can often be unreliable (Flanagan *et al.*, 2010).

One class of variants where functional prediction is much more straightforward is that which truncates the resulting protein product (stop-gain, *frameshift coding, splice disrupting*). The functional prediction is made based on the translational consequence of a mutation against a reference transcript set, for example, Gencode. Protein truncating variants (PTVs) are typically subject to nonsense-mediated decay (NMD), a cellular mechanism that detects nonsense mutations and prevents the expression of truncated proteins, resulting in loss of function of that copy of the protein. To a first approximation, PTVs are thus likely to have the same functional consequence, namely, loss of function (though see below for a more nuanced treatment), so that variants in this class within a gene may naturally be combined in assessing their effect on a trait or phenotype of interest. A second reason for focussing on PTVs is that there is ample evidence from studies of Mendelian phenotypes, common diseases and quantitative traits (Chen *et al.*, 2011; Herman *et al.*, 2012; Jones *et al.*, 2009; Pollin *et al.*, 2008) that PTVs play an important role in the genetic architecture of human traits. Recent studies have highlighted and characterized PTVs (Cohen *et al.*, 2005; Hofker, 2010; Isidor *et al.*, 2011; MacArthur *et al.*, 2012; Musunuru *et al.*, 2010; Nejentsev *et al.*, 2009; Rivas *et al.*, 2011; Schonfeld, 2003), commonly referred to as ‘loss of function’ (LoF) variants (MacArthur *et al.*, 2012). These studies describe the effect and frequency of PTVs: they can have strong effects on phenotype, and they are, in general, rare, as selection has played a role in preventing them from reaching a high frequency in the population (MacArthur *et al.*, 2012).

Here, we introduce a new statistical method for assessing association between PTVs and quantitative traits. We first consider the straightforward case in which all PTVs are assumed to have the same effect, which would be appropriate, for example, if all caused loss of function. We then extend this approach to allow for the possibility that some PTVs have one effect and some have another, for example, if most PTVs are subject to NMD, and hence are LoF, whereas some (e.g. in the final exon) escape NMD and can act as loss of function, gain of function (by deleting an inhibitory domain) or neutral variants. The approach could be extended to allow more than two groups of PTVs, with PTVs in each group having the same effect, but for definiteness we focus in this article on the case of either one or two groups.

We study the properties of the new method and compare it with other approaches. A simple alternative to our similar effects model is to use the mean trait value of the PTV carriers as the test statistic [see e.g. the collapsing methods of Li and Leal (2008)]. Other methods in our comparisons include the sequence kernel association test (SKAT) (Wu *et al.*, 2011), SKAT-O (Lee *et al.*, 2012) and multiple linear regression. SKAT is a variance component test that allows variants with different direction of effects and maintains power when variants in a unit are non-causal, but is less powerful than the collapsing methods for variants with similar effects; SKAT-O uses the data to optimally combine the collapsing test and the non-collapsing SKAT, but pays a penalty for combining multiple methods in a single test (Lee *et al.*, 2012). These methods have been described and compared in Bacanu *et al.* (2012) and Basu and Pan (2011). The main difference between our methods and the previously existing ones is that our Bayesian model comparison framework automatically incorporates information about the factors affecting power of the study (such as the number of PTV carriers). We discuss how this affects the ranking of genes within a study.

We apply our methods to unpublished sequence and exome-single nucleotide polymorphism (SNP)-array data and find a strong signal at *APOC3* where PTVs lead to decreased plasma triglyceride levels.

## 2 METHODS

Assume that among *N* individuals studied, *n* individuals carry one of the *k* PTVs observed in a gene considered, typically 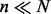. Because PTVs are typically rare, we will assume here that individuals carry at most one such variant. (Two PTVs on the same chromosome are likely to have the same effect as one PTV, and individuals with PTVs on both chromosomes could be treated either as a separate class or in the same class if a dominant model were thought appropriate.) The same PTV might be carried by several individuals.

We denote by 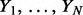 the standardized quantitative trait values of the individuals and assume that the trait values 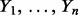 correspond to the carriers of PTVs and the values 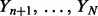 correspond to the non-carriers of PTVs. We further assume that standardized trait values across the whole sample follow a standard normal distribution. If necessary this can be achieved by applying quantile normalization. If the PTV has a big effect on the trait, individuals carrying PTVs might have extreme trait values, which would be moved closer to the other values under quantile normalization. In this setting, there is a potential loss of power through quantile normalization, but we see this as being outweighed by the additional robustness it provides. Outlying observations can occur for many reasons aside from genetics (typically measurement errors), and the individuals who have these outlying observations will have PTVs at some genes, so that in the context of a genome-wide analysis, failure to quantile normalize can lead to a strong (false-positive) signal at the genes where the individuals with the outlying observations have a PTV. Analogous issues arise in Genome-wide association studies (GWAS) for quantitative traits where individuals with extreme trait values can contribute to a strong signal at SNPs where they are called homozygous for a rare variant.

Under the null model, the gene does not affect the trait, and hence the trait values among both the PTV carriers and the PTV non-carriers follow the standard normal distribution:

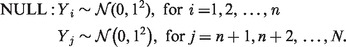

The statistical challenge is then in principle straightforward: one looks for (strong) evidence against the null hypothesis. In effect, this reduces to asking whether 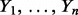 follow a standard normal distribution. (If PTVs at the gene under consideration do affect trait values, this will also cause a deviation from normality for 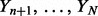, when the entire sample is quantile normalized together, but because 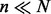, this effect will be small, and we only look for evidence of departure from the null at 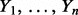.)

One could adopt either a frequentist or a Bayesian statistical approach to the problem. We consider both, but we see substantial advantages here in adopting a Bayesian framework, for reasons we return to below.

The Bayesian approach requires specification of the alternative hypothesis. We first consider the setting, which we call the *similar effects model* (SEM), where all PTVs have the same effect on gene function, and hence on the distribution of trait values.

### 2.1 Similar effects model

Under the SEM, we assume that the effect of a PTV is to shift the mean of the distribution of trait values, so that the trait values of the PTV carriers follow a normal distribution with mean μ and standard deviation *s*, whereas the trait values for the remaining individuals follow a standard normal distribution:

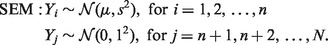

In this work, we fix the value of *s* = 1, but more general approaches could also allow a change under the alternative hypothesis in the variance of trait values, or potentially in their distribution. A change in variance could be handled easily if a conjugate prior were used; because the number of individuals with PTVs will typically be small, power to detect a change in the distribution of trait values will be limited.

**Fig. 1.**
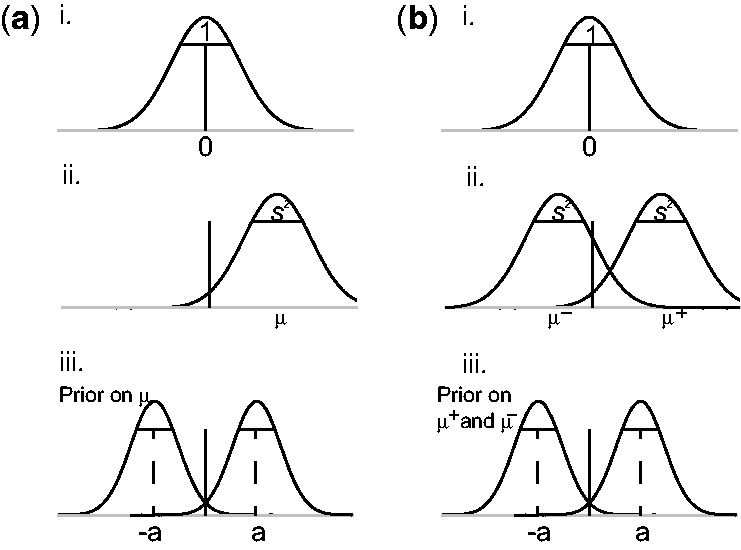
(**a**) Prior and sampling distribution for the SEM: (i) distribution of the trait values under the null model, 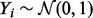; (ii) distribution of the trait values under the alternative model, 
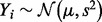
; and (iii) 50:50 mixture of two normal distributions as prior for 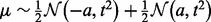. (**b**) Prior and sampling distribution for the GEM: (i) distribution of the trait values under the null model; (ii) under the alternative model, trait values are grouped around 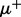 and 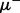; and (iii) priors for 
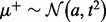
 and 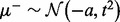

To complete the specification of the alternative model, we must specify the distribution of the trait mean μ under the alternative hypothesis. Because it will not usually be known in advance whether PTVs will increase or decrease trait values, we use a 50:50 mixture of two normal distributions as a prior for μ:

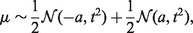



We fix the hyperparameters to 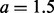 and 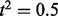. With these values, 95% of the prior mass for μ lies in the set 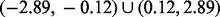.

In a Bayesian framework, the appropriate way to compare the null and alternative models is via the *Bayes factor* (BF) defined as the ratio of the probability densities of the observed data under the two models. In our setting, the BF reduces to a ratio of densities involving only trait values of the carriers of PTVs (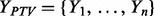) because under both models the data on the non-carriers of PTVs have the same distribution.

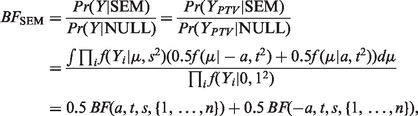

where 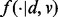 is the density function of the normal distribution with mean *d* and variance *v* and

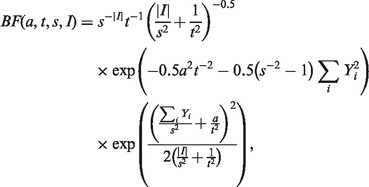

where the index *i* runs through the index set *I* whose size is 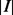.

### 2.2 Grouped effects model

In some biological contexts, different PTVs may have different effects on the trait. For example, different PTVs can affect different transcript isoforms, either trigger or escape NMD, or act as dominant gain of function variants, all of which have been observed in genes predisposing to cancer (Futreal *et al.*, 2004; Isidor *et al.*, 2011; Ruark *et al.*, 2013). To cater for this setting, we extend our alternative model by allowing different groups of PTVs to have different impacts on the trait.

Analysis of RNA sequencing data confirms that transcripts with premature termination codons 50 bp upstream of the final exon–exon junction escape NMD (Geuvadis RNA-Sequencing Project, unpublished data). The mutant mRNA products may well lead to resulting truncated proteins that act in a loss of function, or gain of function manner as observed in *NOTCH2* (Isidor *et al.*, 2011) or have no impact on a trait. A prudent thing to do is to have an alternative model where PTVs >50 bp upstream of the final exon–exon boundary could be placed in one of the groups with all other PTVs in the other group. However, the limitation in this setting is that it may well be the case that variants that escape NMD have a similar effect on trait values as variants that trigger NMD, or have no effect. We extend our alternative model and use biologically informed weights to reflect this:

Let *G* be the set of all 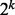 possible assignments of the *k* PTVs into two groups labelled + and −. For any grouping 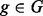, let 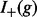 and 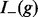 be the sets of indexes of the individuals who carry PTVs that *g* assigns to groups + and −, respectively. We define the model

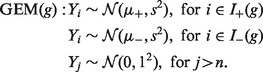

Our priors are 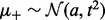 and 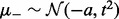, with 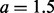 and 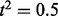, and again we keep *s* = 1 fixed. In other words, we assume that the phenotype of an individual with a truncating variant in a relevant gene will be towards the tails of the distribution.

The BF between 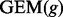 and the null model is

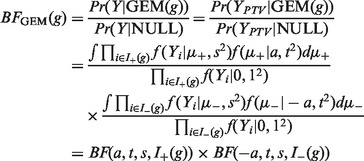



We define the final 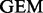 as a linear combination of submodels 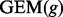 by choosing a weight 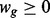 for each grouping 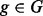 with the constraint 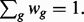 The corresponding BF is

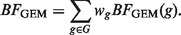



Note that by giving a weight of 0.5 to each of the two groupings in which all variants belong to the same group, and a weight of 0 to all other groupings, we are back to the SEM.

We refer to the approach that uses biologically informed weights based on NMD predictions as grouped effects model (GEM)-NMD. Let 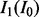 be the indexes of individuals carrying variants that are predicted to trigger (escape) NMD. We define model GEM-NMD by using 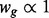 for such groupings 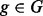 in which individuals in *I*_1_ and individuals in *I*_0_ belong to different groups and by using 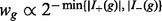 for other groupings.

In sequencing datasets even for current large experiments, the numbers of PTVs in any gene will tend to be small, meaning that there are only few data for distinguishing the null and alternative models. Use of the GEM requires averaging over submodels corresponding to different assignments of PTVs to groups. In the absence of good biological information (such as escape or not from NMD as just described) to assign PTVs to groups, or equivalently to heavily constrain the prior on submodels, the averaging across submodels is likely to come at a considerable cost in power in these settings. In our simulation study and application below, we thus focus only on GEM-NMD, rather than the more general versions of the GEM model.

Finally, we note that under GEM-NMD the averaging over submodels described above maintains the symmetry in SEM: under the prior, the probability that a particular group of PTVs increases trait values while the other group decreases them is the same as the probability that the first group decreases trait values and the second increases them. This is appropriate biologically because it will typically not be possible to predict in advance, for example, whether loss of function mutations will cause an increase or decrease in trait values. Figure 1 provides an illustration of the prior and sampling distribution for SEM and GEM.

### 2.3 Other approaches

When expecting similar effects across the variants, a simple alternative to our SEM is to use 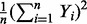 as a test statistic (with 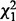 distribution under the null). We call this approach Collapse because it is similar in spirit to the collapsing methods of Li and Leal (2008). Other methods that we use in comparisons are the SKAT (Wu *et al.*, 2011), with the default weight parameters of Beta(1,25) for the linear weighted kernel and Davies’ method to compute the *P*-value, SKAT-O (Lee *et al.*, 2012) using method = ‘optimal’ and multiple linear regression (lm) as implemented in R. Our method and Collapse use the normalized trait values only from the PTV carriers; other methods use trait values on all individuals.

## 3 RESULTS

### 3.1 Simulation study

#### 3.1.1 Comparison of SEM and **C**ollapse

For a fixed value of *n*, both *P*-value from Collapse and 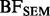 (with 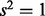) depend on the data only through 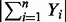, and both show monotonically increasing deviation from the null model with increasing 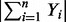. It follows that SEM (with 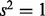) shares the frequentist properties of Collapse, i.e. has the same power and produces the same *P*-values under the null if used as a test statistic. However, a main difference between the methods is that this similarity does not hold across genes with different numbers of PTVs. Rankings across all genes in a study by BF and by *P*-value will in general be different, and for the reasons given in the Discussion, ranking by BF is preferable.

**Fig. 2.**
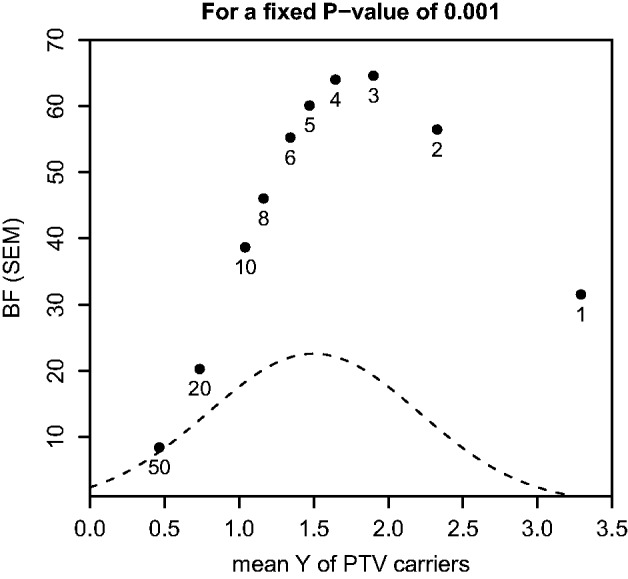
Plots of mean trait value for PTV carriers (*X*-axis) and BF from SEM (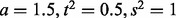) (*Y*-axis), for different values of *n* (written next to each point) the number of PTV carriers, for a fixed *P-*value of 0.001. The dashed line shows the density of the prior (restricted to positive values) on mean effect size under SEM (not on scale)

Figure 2 provides an illustration of this effect. In it, the *P*-value is fixed at 0.001, and the figure plots, for different values of *n*, both the mean value of the trait among PTV carriers and the BF. Because the *P*-value is fixed, any gene with this combination of *n* and mean trait value would have the same *P*-value and hence the same rank in the *P*-value-based ranking. The ranking by BF is lower for small *n* and for larger *n*, compared with intermediate values of *n*. When *n* is small (say 1 or 2), there are few observations of traits for PTV carriers, so limited power to assess the null and alternative hypotheses. To achieve a small *P*-value, these observations need to be relatively large in absolute value, but the Bayesian approach will downweight them because of the limited amount of data, and the prior assumption that changes of that magnitude in trait values for PTV carriers are unlikely. The relative downweighting in the Bayesian approach of datasets with large *n* is for a different reason and is related to Lindley’s paradox in statistics (Lindley, 1957). For example, with *n* = 50, the data correspond to a rather small change in mean trait values. The Bayesian approach compares the probability of this under the null (closely related to the *P*-value) with the probability under the alternative, and it turns out that this small change is also relatively unlikely under the alternative (because the alternative assigns most of its mass to larger changes in trait value), reducing the BF.

#### 3.1.2 Critical values when the BF is used as a test statistic

For ease of use, we provide a table of critical values of the BF when it is used as a frequentist test statistic. As mentioned previously, we are able to evaluate the null distribution of 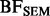 (with 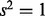) analytically, and hence to calculate critical values analytically. For GEM-NMD, the same can be done by simulations. Table 1 gives the critical value for the BF corresponding to the type I error rates (α) of (i) 0.01, (ii) 0.001 and (iii) 0.0001, evaluated at different numbers of observed PTVs (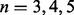).

**Table 1.** Critical values for BFs from SEM and GEM-NMD corresponding to different Type I error rates (α) with 3, 4 and 5 PTVs

α	NPTV	SEM	GEM-NMD^a^
0.01	3	8.765	9.34 (9.30,9.39)
	4	7.77	8.93 (8.88,8.98)
	5	6.81	8.36 (8.32,8.41)
0.001	3	64.56	59.94 (59.01,60.84)
	4	63.98	63.99 (62.76,65.01)
	5	60.07	63.81 (62.63,64.94)
0.0001	3	438.45	362.10 (349.11,380.70)
	4	489.86	438.52 (412.02,460.90)
	5	496.85	459.96.72 (434.96,484.73)
0.00001	3	2814.95	2035.51 (1790.69,2277.75)
	4	3564.12	2703.12 (2336.41,3197.85)
	5	3924.31	3199.53 (2778.93,3765.76)

*Note*: ^a^A total of 10 000 000 replicates were generated to evaluate critical values at the corresponding Type I error rate together with its 95% confidence intervals (L95, U95). The critical values for SEM were calculated analytically.

#### 3.1.3 Power comparisons

We performed power comparisons among the various frequentist approaches, including the use of the BFs described above as frequentist test statistics.

First consider the scenario when all PTVs at a gene have the same effect on the trait, as would be expected for typical ‘loss of function’ variants. To assess this, we simulated 3–5 PTVs each seen in a single individual. Trait values for PTV carriers were drawn from an 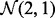 distribution, and the remaining trait values for a total of 2000 trait observations were drawn from an 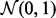. For this scenario (Table 2), the most powerful methods are our SEM and Collapse, which, as explained above, have exactly the same power. SKAT-O (Lee *et al.*, 2012) comes next, and the power is the lowest for SKAT (Wu *et al.*, 2011) and linear regression.

**Table 2.** Power, expressed as a percentage, at 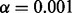, to detect association for two scenarios: (i) variants impact trait values in same direction with similar effects, and (ii) variants impact trait values in different directions with direction of effect determined by NMD

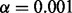	Similar			Grouped		
NPTV	3	4	5	3	4	5
SKAT	42	49	69	44	55	66
SKAT-O	53	65	86	37	45	60
Collapse	55	81	86	2	0	1
SEM	55	81	86	2	0	1
GEM-NMD	—	—	—	51	65	79
SKATw	—	—	—	28	46	56
Multiple linear regression	42	49	69	44	55	66

*Note*: For GEM-NMD, thresholds for the given type I error rate are given in Table 1.

In the second scenario, we assumed that PTVs in a gene contribute to trait variance and can have an effect in opposite directions, with the direction of effect specified by the impact of NMD (Table 2). In our simulation of this scenario, trait values were drawn from an 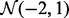 distribution for individuals with a copy of a variant predicted to trigger NMD, and 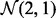 for individuals with a copy of a variant predicted to escape NMD and 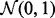 otherwise (one variant was selected to escape NMD). Not surprisingly, GEM-NMD, which uses information on NMD, is clearly the most powerful model. Next comes SKAT and linear regression, then SKAT-O and last SEM and Collapse, which have almost no power in this scenario. In addition, we included a weighted version of SKAT (SKATw) in the power comparisons. For the variants predicted to trigger NMD, we assigned a weight of 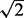, and for the variants predicted to escape NMD, we assigned a weight of 1. This approach ranks below SKAT-O but above Collapse and SEM.

There may be scenarios in which a putative variant is erroneously labelled as a PTV, for example, because of a sequencing artifact or an incorrect annotation. In these settings, our approach will lose power. To illustrate this, we focus on the SEM and simulations applied in Table 2. We assume that one of the 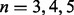 PTVs is actually a sequencing artifact and that the trait value of the corresponding individual is drawn from a standard normal distribution. In this case, power to detect the association decreases to 13,35 and 55% for 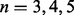, respectively.

### 3.2 Application of the method to plasma triglyceride levels and truncating variant data from the Genetics of Type 2 Diabetes study and Oxford Biobank study

We applied the proposed approach to exome-sequencing data from the Genetics of Type 2 Diabetes (GoT2D) study and plasma triglyceride levels. The dataset consisted of 2760 T2D cases and control individuals of Northern European descent. Exome target capture was performed with the Agilent SureSelect Human All Exon hybrid selection kit and sequence obtained on HiSeq. Subsequent alignment and allele calling used Burrows-Wheeler Aligner (BWA) (Li and Durbin, 2010) and Genome Analysis Toolkit (GATK) (DePristo *et al.*, 2011) generating 688 731 variants that passed quality control and were included in the analysis.

**Fig. 3.**
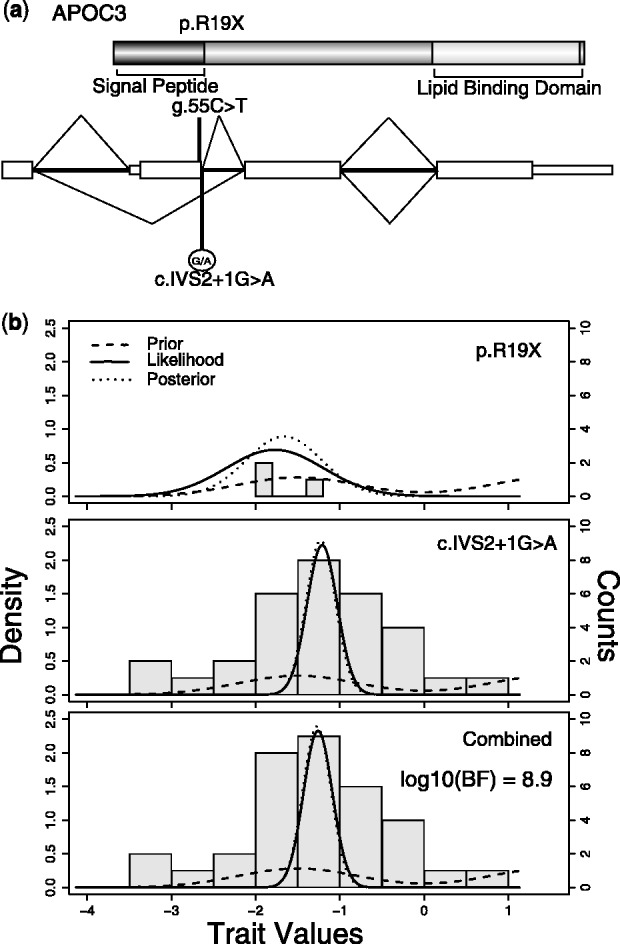
(**a**) Protein and transcript locations of *APOC3* mutations, including predicted impact of splice variant on transcript splicing. Transcript diagram demonstrates that variant *c.*IVS2 + 1G >A will disrupt proper splicing of the second exon and create a new spliced mRNA with exon 1 and exon 3 joining because of proper recognition of splice sequence in the donor site of exon 1 and acceptor site of exon 3. Genomic codon position is shown for the stop-gain mutation, i.e. *g*.55C >T. (**b**) Prior, likelihood and posterior of mean trait value after combining data from the Oxford Biobank study and GoT2D study. The shaded histogram in each panel represents the distribution of trait values for the relevant PTV carriers

To illustrate the performance of our models, we focus here on data for *APOC3* for which a null mutation p.R19X has previously been implicated to be associated to plasma lipid profile and apparent cardioprotection (Hofker, 2010; Pollin *et al.*, 2008). Variant annotation in using the pyplinkseq library (part of PLINK/SEQ toolset [http://atgu.mgh.harvard.edu/plinkseq/] and MAMBA dependency), identifies two candidate PTVs predicted to trigger NMD: the previously mentioned stop-gain mutation, p.R19X (MAF = 0.00025, allele count = 1), and a donor essential splice variant *c*.IVS2 + 1G >A (MAF = 0.002, allele count = 8). After application of the SEM to the data, the posterior distribution indicates that plasma triglyceride levels of individuals with PTVs at *APOC3* are 1.2 standard deviations (SD) lower than the population mean, indicating a strong effect (Fig. 3). The effects of the splice and the stop-gain variants are at 1.19 and 1.44 SD below the population mean, respectively (BF for SEM = 63.44). The splice variant explains 0.29% of the trait variance in our data, whereas the null mutation explains 0.04% of the trait variance. To compare with other methods, we applied (i) Collapse, (ii) SKAT, (iii) SKAT-O and (iv) multiple linear regression to the data (Table 3). We find that our models, Collapse and SKAT-O give similar results (as measured by *P*-value), whereas SKAT and lm give clearly larger *P*-values, probably because they are not assuming similar effect sizes across the two variants. When we compare the ranks of genes in an exome-wide analysis, the SEM and SKAT rank *APOC3* as #3 and #5, respectively.

**Table 3.** Comparison of association *P*-values and BFs for APOC3 truncating variants and plasma triglyceride levels in the GoT2D exome-sequencing study

Measure	Collapse	SKAT	SKAT-O	lm	SEM	GEM-NMD
*P*-value	0.00065	0.0016	0.00063	0.003	0.00065	0.00066
BF	—	—	—	—	63.4	42.3

*Note*: *P*-value for GEM-NMD is based on 1 000 000 simulations and its 95% empirical confidence interval is (0.000607–0.000707).

We were able to confirm these effects using Illumina exome array genotyping in the Oxford Biobank cohort (*n* = 4443 samples, [http://www.oxfordbiobank.org.uk]). Quantification of plasma triglyceride concentrations in the Oxford Biobank was made after an overnight fast. Combined analysis of plasma triglyceride levels and variant genotype data for *c.*IVS2 + 1G >A (allele count = 3) and p.R19X (allele count = 31) from the Oxford Biobank cohort and the GoT2D samples demonstrates strong supporting evidence for protein truncating variant association to low plasma triglyceride levels (BF for SEM = 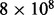). The combined posterior effect size for *c.*IVS2 + 1G >A and p.R19X is 1.23 and 1.66 SD below the mean, respectively.

## 4 DISCUSSION

### 4.1 Application of the method

Larger BFs correspond to stronger evidence in favour of the alternative hypothesis over the null. The BF calculated in our approach has a direct interpretation as the way in which the data changes the weight, which should be given to the null and alternative hypotheses. Formally, the posterior odds (i.e. in the light of the data) on the alternative hypothesis can be calculated by multiplying the prior odds (i.e. before the data) by the BF.

We envisage that a primary application of this and similar methods will be in the analysis of quantitative traits measured in individuals for whom whole-genome or whole-exome sequence data are available, or perhaps for whom genetic data are obtained from an exome-SNP-array.

For a single dataset one could use the size of the BF to indicate the strength of the evidence in favour of the alternative hypothesis. It can also be helpful to plot the posterior distribution on the mean trait value for individuals carrying PTVs.

Many studies will consist of two phases, discovery and replication. In this setting, analyses of the discovery data would aim to rank genes for follow-up according to the evidence for departures from the null hypothesis at that gene. In such a ranking exercise, the use of BFs has considerable advantages over the use of *P*-values. The interpretation of a *P*-value obtained in a particular experiment depends on both the alternative hypothesis and the power of the statistical method used. In the current context, power will change with *n*, the number of PTVs observed at the gene: there will be more power to reject the null hypothesis (for a specific alternative hypothesis) at genes that happen to have more PTVs in the sample, and as a consequence, informally, a low *P*-value will be more informative for a gene with more PTVs than the same *P*-value would be for a gene with fewer PTVs. For this reason, simple ranking of genes by *P*-value is unlikely to be optimal. In contrast, BFs quantify the evidence for the alternative hypothesis as compared with the null (*P*-values only measure tail probabilities under the null), so can be compared naturally even for genes with different numbers of PTVs.

Although we see advantages in adopting a Bayesian perspective, our approach could be used in a frequentist context by calculating the BF and using this as a frequentist test statistic. Conditional on the number of PTV carriers at the gene, *n*, a *P*-value can then be obtained by asking for the probability, under the null hypothesis, of obtaining the same or a larger BF than the one actually calculated from the data. Under the SEM, the null distribution of the BF can be calculated explicitly, allowing a simple analytic computation of *P*-values. For more complicated alternative hypotheses, *P*-values can be obtained easily by simulation under the null.

In the SEM setting, the statistical problem is a simple one, namely, of detecting a mean shift in a (typically small) number of observations. Our method is the obvious Bayesian approach to the problem, and when the resulting BF is used as a test statistic it is effectively equivalent to the obvious frequentist approach. We have, necessarily, used particular prior distributions for the change in mean trait value caused by the PTVs. In our method these are relatively broad, but they could be adjusted as our biological knowledge grows, as could the priors in the GEM model. As more genes are discovered where PTVs affect quantitative traits, the priors could be informed by knowledge of the effect sizes of PTVs at these genes. Two caveats are appropriate here. The first is that early discoveries are likely to correspond to genes where PTVs have larger effects, and the second is that a more precise specification of the prior will gain power if it reflects reality but could substantially lose power otherwise.

What are the sample sizes required to obtain exome-wide significant results? Under the assumption that effect sizes are 2, 1.5 and 1 SDs from the mean, to get a *P*-value of 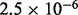 (where 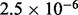 is obtained by Bonferroni correction = 0.05/number of genes), using the SEM would require 8, 14 and 31 PTV carriers, respectively, to achieve 80% power to detect the association. PTV frequencies are likely to vary orders of magnitude between genes, and our current understanding is that only a limited number of genes have a low frequency (>0.1%) PTV, whereas in most genes any single PTV will be private or extremely rare. If a fraction 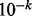 of the population (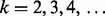) carries a PTV at a particular gene, then we need to sample ∼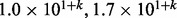 or 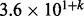 individuals to achieve 80% probability of finding at least 8, 14 or 31 PTV carriers at that gene, respectively.

As we generate more cellular genomics data, we will improve our understanding of the impact of PTVs on gene function. For example, combining DNA and RNA sequence data will enable better predictions of mechanisms underlying NMD, which will, in turn, give more accurate predictions of the biological consequences that the analyzed variants may have. Current annotation of loss of function variants is based on some reference transcript set, for example, GENCODE (Harrow *et al.*, 2012), and selection of variants mapping within the units to include is chosen from the most deleterious annotation across all transcripts. Transcript quantifications for the relevant tissue or cell types will enable better annotation and selection of rare variants for combining in statistical analyses. Thus, now is a good time to think about how this knowledge can be used in future data analyses. Our Bayesian framework provides one way to do this, and we hope that it will stimulate research on both the methodological aspects of these models and on the enhanced prior specification based on biological knowledge.

## 5 CONCLUSION

We have developed and studied a novel statistical method for assessing the association between PTVs and a quantitative trait. The approach is formulated in a Bayesian statistical framework, and can be used in a Bayesian framework or in providing a test statistic for frequentist application. Bayesian application is recommended in ranking genes for follow-up in a discovery dataset. The frequentist application allows calculation of *P*-values for association, which can be helpful in settings where these are required.

We have presented two versions of the method. In the first, SEM, all PTVs are assumed to have effects in the same direction. This would be appropriate, for example, if all PTVs were thought to result in loss of function of that copy of the gene. We also described a more general version of the approach allowing different classes of PTV in terms of their effect on trait values. We see the major application of this more general method in taking a more nuanced view of the consequences of PTVs, and distinguishing between those likely to be subjected to NMD and those that escape NMD (e.g. many or all PTVs in the last exon of the gene) and may result in gain of function. As our biological understanding of the NMD mechanism increases, we expect that it will add power to allow potentially different effects for PTVs escaping NMD, for example, by applying GEM-NMD (in which they may have similar effects or different effects) and SEM (in which their effect on the trait would be the same, even though the molecular consequences of the mutation would be different).

Finally, we have established and replicated an association between PTVs in *APOC3* and lowering of plasma triglyceride levels.
